# Generating Daily Synthetic Landsat Imagery by Combining Landsat and MODIS Data

**DOI:** 10.3390/s150924002

**Published:** 2015-09-18

**Authors:** Mingquan Wu, Wenjiang Huang, Zheng Niu, Changyao Wang

**Affiliations:** 1 The State Key Laboratory of Remote Sensing Science, Institute of Remote Sensing and Digital Earth, Chinese Academy of Sciences, Beijing 100101, China; E-Mails: niuzheng@radi.ac.cn (Z.N.); wangcy508@radi.ac.cn (C.W.); 2 Laboratory of Digital Earth Sciences, Institute of Remote Sensing and Digital Earth, Chinese Academy of Sciences, Beijing 100094, China; E-Mail: huangwj@radi.ac.cn

**Keywords:** spatial and temporal data fusion, remote sensing, MODIS, Landsat, FROM-GLC

## Abstract

Owing to low temporal resolution and cloud interference, there is a shortage of high spatial resolution remote sensing data. To address this problem, this study introduces a modified spatial and temporal data fusion approach (MSTDFA) to generate daily synthetic Landsat imagery. This algorithm was designed to avoid the limitations of the conditional spatial temporal data fusion approach (STDFA) including the constant window for disaggregation and the sensor difference. An adaptive window size selection method is proposed in this study to select the best window size and moving steps for the disaggregation of coarse pixels. The linear regression method is used to remove the influence of differences in sensor systems using disaggregated mean coarse reflectance by testing and validation in two study areas located in Xinjiang Province, China. The results show that the MSTDFA algorithm can generate daily synthetic Landsat imagery with a high correlation coefficient (*R*) ranged from 0.646 to 0.986 between synthetic images and the actual observations. We further show that MSTDFA can be applied to 250 m 16-day MODIS MOD13Q1 products and the Landsat Normalized Different Vegetation Index (NDVI) data by generating a synthetic NDVI image highly similar to actual Landsat NDVI observation with a high *R* of 0.97.

## 1. Introduction

Because Earth surface observations can be obtained periodically by satellite remote sensing, this technology has become a foremost technique for monitoring land surface processes [1]. Since 1978, satellite remote sensing of the land surface process has been dominated by polar-orbiting sensors including the Advanced Very High Resolution Radiometer (AVHRR) [2], Systeme Pour l’Observation de la Terre (SPOT) Vegetation (VGT) [3], and the Moderate Resolution Imaging Spectroradiometer (MODIS) [4]. The temporal resolution of these coarse resolution sensors is one–two days, which means they can image the entire Earth at one–two-day intervals. Owing to their high temporal resolutions, the time series data of these sensors are widely used in land surface processes dynamic monitoring applications [5,6,7,8] such as land cover and land change [9,10], crop mapping and production forecasts [11,12], disasters such as fires [13], floods [14], and algal blooms, and forest [15] and grassland [16] ecosystems. However, these high temporal resolution data have low spatial resolution; therefore, the signals recorded by these sensors are not suitable for highly spatially variant land surface processes monitoring. Medium spatial resolution sensors such as the Thematic Mapper (TM), Enhanced Thematic Mapper Plus (ETM+), and Operational Land Imager (OLI) sensors on Landsat satellites are other types satellite data widely used in land surface monitoring for applications such as detailed land use and land cover mapping [17,18], environmental monitoring [19], and ecological system dynamic monitoring [20,21]. However, these data have low temporal resolution and cloud interference; therefore, their application to land surface monitoring leads to a shortage of valid data. Leckie [22] found that the probability of acquiring cloud-free Landsat images for a given time with cloud cover <10% can be as low as 10%. Therefore, no single satellite can provide data to meet the challenges of high spatial and temporal land surface process monitoring.

To address this problem, several spatial and temporal data fusion approaches have been proposed to generate high spatial and temporal data by fusing coarse and medium spatial resolution data. Gao *et al.* [23] introduced the spatial and temporal adaptive reflectance fusion model (STARFM) for blending MODIS and Landsat imagery, and Roy *et al.* [24] used a semi-physical fusion approach to fuse multi-temporal MODIS–Landsat data. Several studies have applied the STARFM to urban environmental variable extraction, vegetated dry-land ecosystem monitoring, public health studies, and daily land surface temperature generation [25,26,27,28,29]. Hilker *et al.* [25] improved the STARFM for the spatial temporal adaptive algorithm for mapping reflectance change (STAARCH) for producing synthetic imagery and detecting changes. Zhu *et al.* [30] enhanced the STARFM for complex heterogeneous regions. Emelyanova *et al.* [31] assessed the accuracy of STARFM and ESTARFM in two landscapes with contrasting spatial and temporal dynamics. However, most of these models are based on the assumption that the change in reflectance *r* of each land cover class is linear, which is not accurate for land cover types such as vegetation [32].

In response, other scholars have proposed methods based on a linear mixed model that assumes that the reflectance of each coarse spatial resolution pixel is a linear combination of the responses of each land cover class contributing to the mixture [33,34,35]. However, owing to differences in environmental factors such as altitude, morphology, and soil type or management factors such as sowing date and fertilization, this assumption is not always valid. Zhukov *et al.* [36] and Maselli [37] addressed this problem by using the neighboring pixel information based on the assumption that spectral properties of a land cover class do not show great variations in the surrounding pixels. On the basis of their work [36,37], Busetto *et al.* [32] developed a new method in which the neighboring pixels are selected and weighted on the basis of their Euclidean distances from the target pixel; this method also considers the spectral similarity of the subcomponents with those of the targets. However, the reflectance disaggregated by these proposed methods is the mean reflectance of each land cover class in the identified subset *s*, which is still not equal to the real surface reflectance *r* of fine-resolution pixels. To solve this problem, Wu *et al.* [38] proposed a spatial and temporal data fusion approach (STDFA) based on the assumption that the temporal variation properties of each fine pixel in the same class are constant. They applied this method to the estimation of the high spatial and temporal resolution land surface temperature [39] and leaf area index [40]. They also validated that ESTARFM and STDFA can be applied to combine Huanjing (HJ) charge coupled device (CCD) and MODIS reflectance data together with Gaofen satellite no. 1 (GF-1) wide field of view camera (WFV) and MODIS reflectance data [41]. Gevaert and García-Haro [42] introduced an unmixing-based algorithm and compared it with STARFM. They recommended using unmixing-based data fusion for situations in which the spectral characteristics of the medium-resolution input images are downscaled.

However, STDFA has several limitations. The differences in sensor systems are not considered, and the window sizes used to select coarse pixels involved in the solution of the linear mixed models is fixed. The best window sizes for different land cover classes may vary according to the different spatial distribution of each land cover class; a constant window size may result in lower accuracy solution of the linear mixed models of some land cover classes. To address these limitations, the objectives of the present study are (1) to modify STDFA by introducing sensor difference correction and adaptive window size selection methods; (2) to test and analyze the applicability of the modified spatial and temporal data fusion approach (MSTDFA) in other data such as MODIS MOD13Q1 products and Landsat Normalized Different Vegetation Index (NDVI) data; and (3) to test and analyze the availability of Finer Resolution Observation and Monitoring of Global Land Cover (FROM–GLC) data in MSTDFA.

## 2. Methods

### 2.1. Method Inputs and Processing Steps

**Figure 1:**
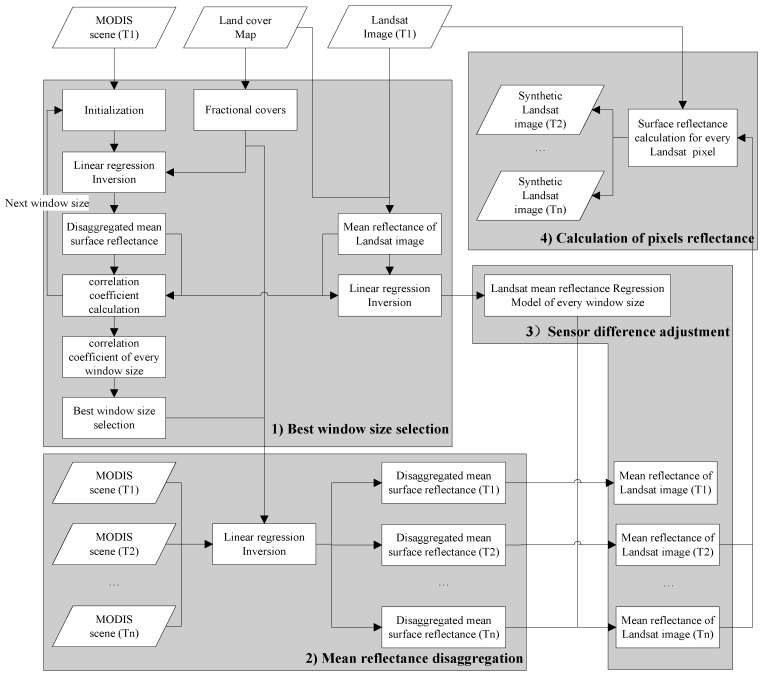
Flowchart of the modified spatial and temporal data fusion approach (MSTDFA) algorithm. The processing steps of the three main blocks are explained in Section 2.2, Section 2.3, Section 2.4 and Section 2.5.

To address the weaknesses of STDFA in the estimation of daily synthetic Landsat imagery, MSTDFA is proposed in this paper. The input of this algorithm includes a Landsat image, land cover and time series MODIS reflectance data that were acquired on the same day as was the Landsat image, and at least one MODIS reflectance dataset acquired on the same day as was the Landsat image selected for simulation. The output of this algorithm is a time series of synthetic Landsat imageries in which the acquisition date can be considered the same as that of the time series MODIS data. The algorithm includes four steps: (1) best window size selection; (2) mean reflectance disaggregation; (3) sensor difference adjustment; and (4) calculation of each pixel’s reflectance and outputting of the daily synthetic Landsat imagery. A flowchart of the algorithm is shown in Figure 1. The algorithm is run by one band. To generate a multispectral synthetic Landsat imagery, we need to apply this method for each band, respectively.

### 2.2. Selecting the Best Window Size

According to the unmixing theory, the reflectance of a coarse-resolution spatial pixel is assumed to be a linear combination of the responses of each land cover class contributing to the mixture [33]. The coarse spatial reflectance *R* (*i*, *t*) of the landscape thus consists of k discrete land cover class *c* weighted by their class fractional cover as

(1) $$R(i,t_{i})=\sum_{c=0}^{k}f_{c}(i,c)\times {\bar{r}}_{f}(c,t_{i})+\xi (i,t_{i})$$

Constrained: $\sum_{c=0}^{k}f_{c}(i,c)=1$ and $1\geq f_{c}(i,c)\geq 0$ for all, where $f_{c}(i,c)$ is the fractional cover of class *c* in coarse pixel *i*, which is usually assumed to not change over time; ${\bar{r}}_{f}(c,t_{i})$ is the mean reflectance of fine-resolution homogeneous pixels belonging to land cover class *c* at time *t_i_*; and $\xi (i,t_{i})$ is the residual error term. If we know the coarse spatial reflectance *R* (*i*, *t_i_*) and the fractional cover values, Equation (1) can be solved with the ordinary least squares technique and by generating the disaggregated mean surface reflectance value ${\bar{r}}_{f}(c,t_{i})$ for class *c* at time *t_i_*. Generally, the fractional cover values were extracted from high-resolution spatial land cover map.

Then, by inputting the fractional cover values and the time series coarse spatial reflectance from time *t_1_* to time *t_n_*, the time series mean surface reflectance value ${\bar{r}}_{f}(c,t_{i})$ for each class was calculated by solving Equation (1) using the ordinary least squares technique. Since this solved mean surface reflectance was disaggregated by the ordinary least squares technique, we defined this disaggregated mean surface reflectance as ${\bar{r}}_{d}(c,t_{i})$ to distinguish with the mean surface reflectance ${\bar{r}}_{f}(c,t_{i})$ calculated from real fine-resolution pixels. The time series mean surface reflectance value ${\bar{r}}_{f}(c,t_{i})$ for each class ranges from 0 to 1. Disaggregated mean surface reflectance out of this constraint were not used to build a linear model between the disaggregated mean surface reflectance and actual TM mean surface reflectance for the adjustment of sensor difference. To reduce the influence of spatial variation and geolocation errors, the solution of Equation (1) was conducted in a rectangular window centered to the MODIS target pixel. Owing to the different spatial distribution of each land cover class, the best window size s for each land cover class may be different. To determine the best window size *s* for each land cover class, rectangles of lengths of 3–120 MODIS pixels for different classes were tested. Firstly, for window length *l*, a subset *s* of MODIS pixels centered to the MODIS target pixel *m_k_*, fractional cover data, and Landsat pixels was extracted. Then, the disaggregated mean surface reflectance value ${\bar{r}}_{d}(c,t_{i},m_{k})$ and real mean surface reflectance value of Landsat pixels ${\bar{r}}_{f}(c,t_{i},m_{k})$ for the MODIS target pixel *m_k_* were calculated. Thirdly, along with the MODIS target pixel being moved to cover the entire MODIS image, a real mean fine reflectance vector ${\bar{r}}_{f}\left(c,t_{i},m\right)$ and a disaggregated mean coarse reflectance vector ${\bar{r}}_{d}\left(c,t_{i},m\right)$ pair for land cover class *c* was calculated to allow calculation of the correlation coefficient between those two vectors for window length *l*. ${\bar{r}}_{f}\left(c,t_{i},m\right)$ and ${\bar{r}}_{d}\left(c,t_{i},m\right)$ were described as follows:

(2) $${\bar{r}}_{f}\left(c,t_{i},m\right)=({\bar{r}}_{f}\left(c,t_{i},m_{1}\right),{\bar{r}}_{f}\left(c,t_{i},m_{2}\right),\cdots ,{\bar{r}}_{f}\left(c,t_{i},m_{n}\right))$$

(3) $${\bar{r}}_{d}\left(c,t_{i},m\right)=({\bar{r}}_{d}\left(c,t_{i},m_{1}\right),{\bar{r}}_{d}\left(c,t_{i},m_{2}\right),\cdots {\bar{r}}_{d}\left(c,t_{i},m_{n}\right))$$

where *m*_1_, *m*_2_, *and m_n_* is the MODIS target pixel; *n* is the number of the MODIS pixel. Obviously, the best window length *l* for land cover class *c* will have the highest correlation coefficient. Thus, the window length *l* with the highest correlation coefficient *R* was set to the best window length for land cover class *c*.

### 2.3. Disaggregating Mean Reflectance

After the best window size for land cover class *c* is determined, the mean reflectance for land cover class *c* of target MODIS pixel *m_k_* can be calculated by solving Equation (1) using the ordinary least squares technique by inputting a subset *s* for window length *l* of the MODIS pixels centered to the MODIS target pixel *i*, fractional cover data, and Landsat pixels.

### 2.4. Adjusting Sensor Difference

Owing to sensor system differences in bandwidth, acquisition time, spectral response functions, geolocation errors, and atmospheric correction, there is a need to adjust the disaggregated mean reflectance ${\bar{r}}_{d}(c,t_{i})$ for land cover class *c* to the real mean fine reflectance ${\bar{r}}_{f}(c,t_{i})$. In Section 2.2, a real mean fine reflectance vector ${\bar{r}}_{f}\left(c,t_{i},m\right)$ and disaggregated mean coarse reflectance vector ${\bar{r}}_{d}\left(c,t_{i},m\right)$ pair for each land cover class was generated. This allowed for construction of a linear model between the real mean fine reflectance vector and disaggregated mean coarse reflectance vector by using linear regression analysis, which can be described as:

(4) $${\bar{r}}_{f}(c,t_{i},m)=a\times {\bar{r}}_{d}(c,t_{i},m)+b$$

where a and b are coefficients of the linear regression model. Then, this model was used to calculate real time series mean fine reflectance ${\bar{r}}_{f}(c,t_{i})$ from time *t_2_* to time *t_n_* from the time series disaggregated mean coarse reflectance ${\bar{r}}_{d}(c,t_{i})$.

### 2.5. Calculating Pixels Reflectance and Method Outputs

Since the time series mean fine reflectance ${\bar{r}}_{f}(c,t_{i})$ from time *t_1_* to time *t_n_* was calculated, the time series reflectance of each fine resolution pixel can be determined using the SRCM model proposed by Wu *et al.* [38], which is described as

(5) $${\bar{r}}_{f}(c,t_{i})-{\bar{r}}_{f}(c,t_{1})=r_{f}(c,t_{i},k)-r_{f}(c,t_{1},k)$$

where ${\bar{r}}_{f}(c,t_{i})$ and ${\bar{r}}_{f}(c,t_{1})$ is the mean fine reflectance at time *t_i_* and *t_1_*, $r_{f}(c,t_{i},k)$ and $r_{f}(c,t_{1},k)$ is the reflectance of pixel *k* of class *c* in target MODIS pixel *m_k_* at time *t_i_* and *t_1_*. With the ${\bar{r}}_{f}(c,t_{1})$ and $r_{f}(c,t_{1},k)$ obtained from the Landsat scene at the time *t_1_* and time series ${\bar{r}}_{f}(c,t_{i})$ calculated in Section 2.4, MSTDFA allows the output of time series synthetic 30 m Landsat imagery.

## 3. Method Tests and Results

### 3.1. Study Area

**Figure 2:**
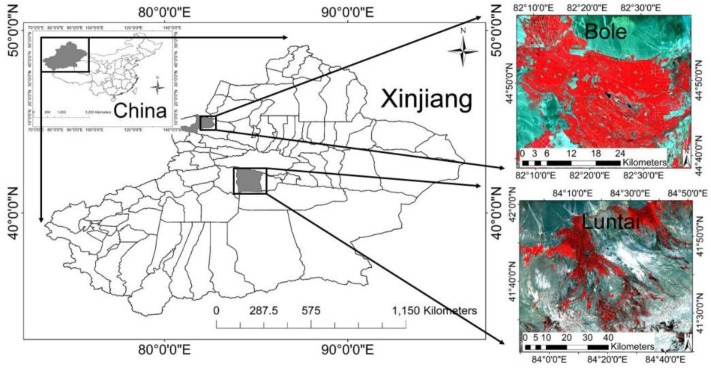
Locations of the study areas.

Two study areas located in Xinjiang Province, West China, were selected to test and validate this method (Figure 2). The first is Bole County, Xinjiang Province, China, located in the valleys between Alatau and Gang Giger mountains, Boertala River. The area to the west of Bole is mountainous, whereas that to the east is plains. In the plains area, the main land use type is farmland, in which the crop plots are usually large. Therefore, the land cover types in this area are relatively homogeneous. The second study area is Luntai County, Xinjiang Province, China, located in southern Tianshan, northern Tarim Basin. The areas north of Luntai are hills, whereas the middle and the southern parts are plains. The crop plots in Luntai are very small; thus, the landscapes are heterogeneous.

### 3.2. Data and Pre-Processing

#### 3.2.1. Landsat Data and Pre-Processing

Three Landsat-5 TM datasets in Bole and Three Landsat-8 OLI datasets in Luntai were used in this study (Table 1). All data were acquired in clear sky conditions and were provided by the United States Geological Survey (USGS). The Landsat data used in the Bole study area were surface reflectance products, whereas those used in Luntai were Level L1T products. The three Landsat L1T products were atmospherically corrected by using the Fast Line-of-Sight Atmospheric Analysis of Spectral Hypercubes (FLAASH) Atmospheric Correction Model in software ENVI 5.0. Then, the six Landsat datasets were georeferenced by using a second-order polynomial warping approach based on the selection of 43 ground control points (GCPs) using a 1:10,000 topographic map by the nearest neighbor resampling method with the position error within 0.74 Landsat pixels.

**Table 1:** Satellite images used in this study.

Study Area	Landsat-5 TM/ Landsat-8 OLI			MODIS	
	Acquisition Date	Path/Row	Usage	Acquisition Date	Usage
Bole	11 July 2011	146/29	Reference Classification	12 July 2011	Mean reflectance estimation
	27 July 2011	146/29	Validation	28 July 2011	
	13 September 2011	146/29	Validation	14 September 2011	
Luntai	4 September 2013	144/31	Validation	3 September 2013	Mean reflectance estimation
	6 October 2013	144/31	Reference Classification	7 October 2013	
	22 October 2013	144/31	Validation	21 October 2013	

Landsat images acquired on 11 July 2011 in Bole and on 6 October 2013 in Luntai were used as reference images for building a linear model between Landsat and MODIS mean reflectance and to calculate the reflectance of fine pixels from the mean reflectance. These images were also used for land cover mapping, which is explained in Section 3.2.3. The subsequent Landsat images were used to evaluate this algorithm.

#### 3.2.2. MODIS Data and Pre-Processing

Six daily MODIS surface reflectance products (MOD09GA, 500 m) obtained in clear-sky conditions were used in this study (Table 1). Ideally, the MODIS image acquisition date should be the same as the acquisition date of Landsat data. However, the quality of MODIS data acquired on the same date as the Landsat data was not good in Bole and Luntai; therefore, these MODIS data were replaced with data of good quality acquired one day earlier or later than the Landsat data.

These six MODIS images were reprojected from the native Sinusoidal projection to a UTM-WGS84 reference system and were resized to the selected study area using MODIS Reprojection Tool (MRT) software. We also resized the spatial resolution from 500 m to 480 m with a nearest neighbor resampling method in MRT. All of these MODIS data were then georeferenced by a second-order polynomial warping approach based on the selection of 38 GCPs on 480 m Landsat images with a nearest neighbor resampling method in which the position error was within 0.63 MODIS pixels. The 480 m Landsat images were resized from georeferenced Landsat images by using the pixel aggregate resampling method.

#### 3.2.3. Land Cover Data

Two types of land cover data were used in this study. The first was mapped by using the maximum likelihood classification method from the reference Landsat images with 1196 field survey data including 334 plots in Bole and 862 plots in Luntai. The field survey data in Bole and Luntai was obtained in 2011 and 2013, respectively. These land cover data were used in the generation of high spatial and temporal synthetic Landsat imageries. Currently, abundant global and regional information of land cover and use are provided, for example, by National Land Cover Database (NLCD) and FROM–GLC data. Usually these sources will be updated every five years. To test the applicability of these data in MSTDFA, the FROM–GLC data in Bole mapped by using Landsat-5 TM data acquired on 21 July 2009 was used in MSTDFA. The classification accuracy of the FROM–GLC data in Bole was evaluated by using a confusion matrix with regions of interest (ROIs) selected by using visual interpretation methods and field survey data. Table 2 shows the accuracy evaluation results. The overall accuracy and Kappa coefficient of the FROM–GLC data in Bole is 78.06% and 0.65, respectively.

**Table 2:** Accuracy evaluations of Finer Resolution Observation and Monitoring of Global Land Cover (FROM–GLC) data in Bole.

Class	Reference Data							Prod. Acc. (%)	User Acc. (%)
	Water	Forest	Grass	Shrub	Impervious	Cropland	Bare Land		
Water	1615	0	0	0	0	0	0	25.47	100
Forest	0	972	0	0	0	0	0	35.37	100
Grass	79	1775	349	372	824	541	0	27.35	8.86
Shrub	0	0	390	71	0	5	0	8.73	15.24
Impervious	700	0	0	0	2035	0	0	65.9	74.41
Cropland	425	0	470	370	200	25,928	0	96.03	94.65
Bare land	3522	1	67	0	29	525	5669	100	57.77

### 3.3. Results and Accuracy Assessment

#### 3.3.1. Results of Landsat Mean Reflectance Regressing

A linear model between Landsat mean reflectance and disaggregated mean coarse reflectance at time *t_1_* (Bole: 11 July 2011; Luntai: 6 October 2013) was built successfully by using linear regression analysis. Table 3 shows the best window size for each band and each land class. Table 4 shows that high-correlation coefficients *R* were acquired in these two study areas. This result demonstrates that the adaptive window size and moving steps selection method have the ability to select the best window size for the disaggregation of coarse pixels.

**Table 3:** The best window size for each band and each land class.

Bole	Best Window Size (MODIS pixels, 500 m)					
Class	Blue	Green	Red	NIR	SWIR1	SWIR2
Forest	45	37	45	15	33	41
Corn	45	37	45	27	37	37
Cotton	15	23	19	35	45	19
Desert	35	35	39	45	39	39
Bare land	41	45	39	41	45	45
Water	31	23	25	35	37	43
Building land	35	37	37	45	43	13
Other crops	41	45	45	13	45	43
**Luntai**	**Best Window Size (MODIS pixels, 500 m)**					
**Class**	**Blue**	**Green**	**Red**	**NIR**	**SWIR1**	**SWIR2**
Cotton	71	73	73	37	33	21
Water	67	59	65	41	57	57
Building land	57	51	65	75	75	67
Bare land	15	19	75	75	19	39
Desert	63	39	33	37	49	13
Corn	73	73	73	73	71	73

**Table 4:** Linear model built by linear regression analysis between Landsat mean reflectance and disaggregated mean coarse reflectance.

Bole: y=x×b+a																		
	Blue			Green			Red			NIR			SWIR1			SWIR2		
Class	R^2^	a	b	R^2^	a	b	R^2^	a	b	R^2^	a	b	R^2^	a	b	R^2^	a	b
Forest	0.984	0.022	0.972	0.929	0.080	0.389	0.956	0.036	0.775	0.972	0.055	0.745	0.929	0.075	0.658	0.869	0.045	0.761
Corn	0.854	0.018	0.771	0.748	0.022	0.581	0.895	0.011	0.783	0.757	0.087	0.708	0.912	0.091	0.328	0.792	0.046	0.338
Cotton	0.490	0.035	0.205	0.487	0.023	0.700	0.593	0.031	0.519	0.939	−0.039	1.021	0.821	0.025	0.787	0.584	0.041	0.511
Desert	0.966	−0.021	1.364	0.994	0.010	0.974	0.996	−0.005	1.003	0.996	−0.027	1.043	0.990	0.003	0.907	0.996	−0.028	1.015
Bare land	0.980	0.021	0.929	0.988	0.016	0.928	0.996	0.006	0.955	0.994	0.008	1.002	0.996	−0.009	1.014	0.941	−0.083	1.351
Water	0.914	0.027	0.931	0.962	−0.017	1.233	0.986	−0.002	1.046	0.984	0.060	−0.120	0.543	0.022	−0.073	0.642	0.015	−0.035
Building land	0.806	0.037	0.861	0.953	0.001	0.982	0.958	−0.019	1.045	0.984	0.028	0.858	0.918	0.012	0.552	0.824	−0.053	0.925
Other crops	0.953	0.020	0.869	0.958	−0.018	1.059	0.972	−0.011	1.028	0.951	0.068	0.799	0.951	−0.034	0.989	0.958	0.006	0.906
**Luntai:** y=x×b+a																		
	**Blue**			**Green**			**Red**			**NIR**			**SWIR1**			**SWIR2**		
**Class**	**R^2^**	**a**	**b**	**R^2^**	**a**	**b**	**R^2^**	**a**	**b**	**R^2^**	**a**	**b**	**R^2^**	**a**	**b**	**R^2^**	**a**	**b**
Cotton	0.910	0.007	0.831	0.824	0.014	0.763	0.841	0.032	0.678	0.699	−0.018	1.102	0.691	0.056	0.685	0.555	0.082	0.423
Water	0.845	0.146	−0.872	0.839	0.189	−0.475	0.941	0.216	−0.508	0.882	−0.061	0.811	0.852	−0.017	0.456	0.785	0.001	0.358
Building land	0.968	0.023	0.825	0.947	0.038	0.734	0.945	0.060	0.662	0.984	0.142	0.435	0.972	0.112	0.509	0.964	0.072	0.686
Bare land	0.968	0.029	0.902	0.990	0.005	1.013	0.990	0.056	0.836	0.994	0.048	0.873	0.996	−0.007	1.022	0.994	0.006	0.991
Desert	0.962	0.056	0.766	0.937	0.128	0.541	0.916	0.146	0.556	0.964	0.149	0.586	0.750	0.248	0.351	0.676	0.121	0.660
Corn	0.867	−0.011	1.108	0.755	0.011	0.792	0.752	−0.001	0.910	0.870	0.078	0.826	0.627	0.147	0.297	0.585	0.072	0.483

x: disaggregated mean coarse reflectance; y: Landsat mean reflectance.

#### 3.3.2. Results of Synthetic Landsat Image Generation

**Figure 3:**
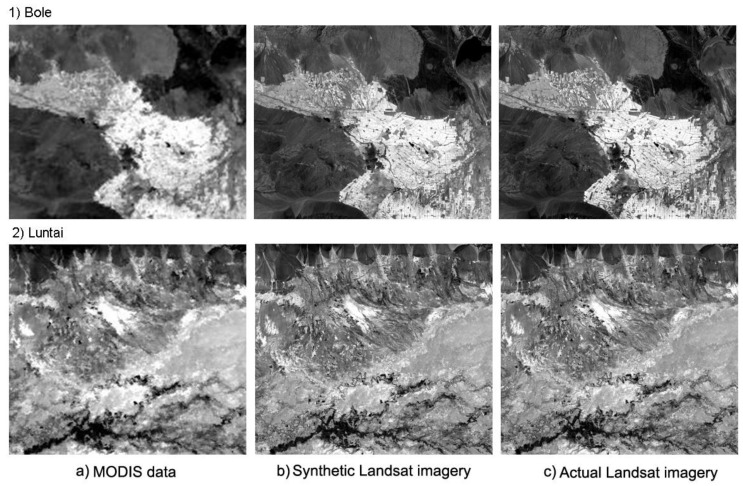
Comparison of Near-infrared (NIR) band surface reflectance data of the Moderate Resolution Imaging Spectroradiometer (MODIS; **left**), synthetic Landsat image (**middle**); and actual Landsat image (**right**) acquired on 11 July 2011 in Bole (upper panels) and 6 October 2013 in Luntai (lower panels), respectively.

By using MSTDFA, four synthetic Landsat images were outputted that contained six bands including blue, green, red, near infrared (NIR), short-wave infrared 1 (SWIR1), and SWIR2. The acquisition date of these data can be considered the same as that for MODIS. Figure 3a shows the actual observation of MODIS surface reflectance on the NIR band acquired on 28 July 2011 in Bole and on 21 October 2013 in Luntai, and Figure 3b shows the synthetic surface reflectance imagery on the NIR band generated by MSTDFA in the two study areas. Figure 3c shows the actual observation of Landsat NIR band surface reflectance acquired on 27 July 2011 in Bole and on 22 October 2013 in Luntai. Through visual interpretation, we determined that the synthetic and actual Landsat data are highly similar and were unable to be distinguished with the unaided eye.

#### 3.3.3. Accuracy Assessment

**Figure 4:**
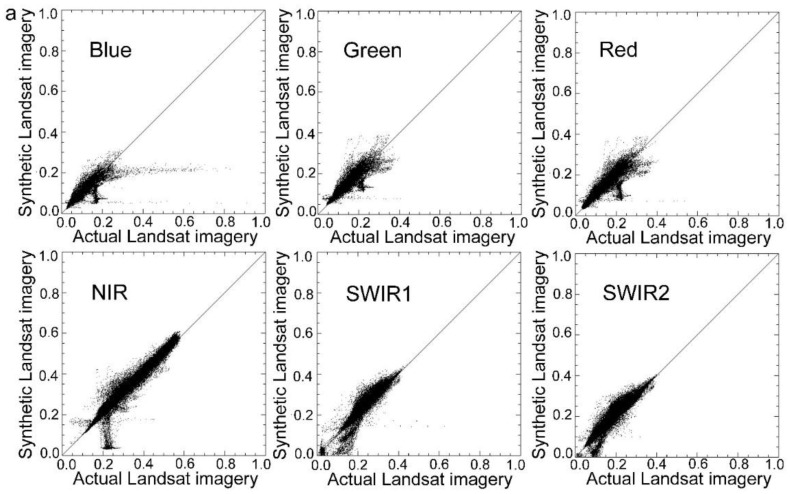

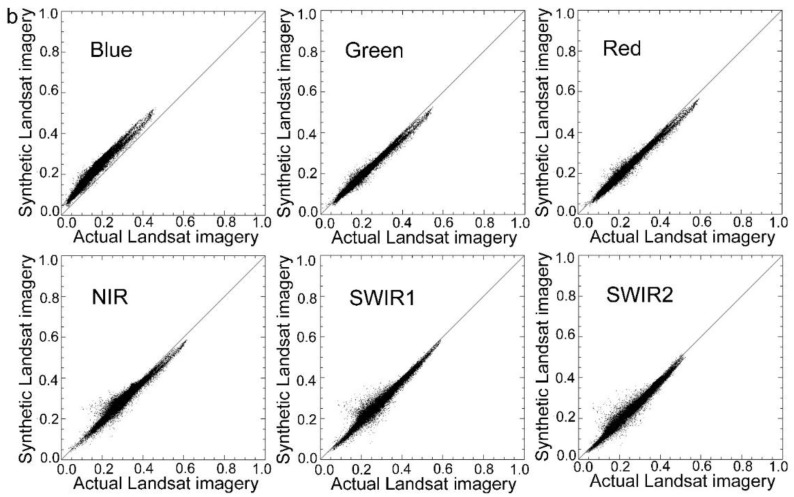
Scatter plots between the actual and synthetic imagers of Landsat at (**a**) Bole and (**b**) Luntai by using the modified spatial and temporal data fusion approach (MSTDFA).

In this study, four actual observations of Landsat data were used to evaluate the accuracy. Closer similarity of the synthetic Landsat image to the actual image relates to the higher precision of the method. To quantitatively evaluate the similarity between the actual observations and synthetic images, correlation analysis was used to calculate the correlation between the synthetic Landsat imagery and the actual observation of Landsat data. Several indicators such as the coefficient (*R*), variance, mean absolute difference (*MAD*), *bias*, and *RMSE* were calculated. Table 5 shows the results of this analysis. As indicated in Table 5, MSTDFA can generate synthetic Landsat images with high similarity to the actual images. Most synthetic Landsat images had a high correlation with the actual Landsat imageries with a coefficient (*R*) higher than 0.90. Figure 4 shows the scatter plots between the actual and synthetic Landsat images, which were close to the 1:1 line. These results indicate a high similarity between the actual and synthetic Landsat data and that MSTDFA has high accuracy in generating synthetic Landsat images.

**Table 5:** Results of correlation analysis between synthetic and actual Landsat imageries.

Study Area	Bole					Luntai				
Date	27 July 2011					4 September 2013				
Parameters	R	Var	MAD	RMSE	Bias	R	Var	MAD	RMSE	Bias
Blue	0.646	0.002	0.011	0.044	−0.001	0.917	<0.001	0.016	0.038	0.029
Green	0.909	0.003	0.012	0.023	−0.003	0.925	<0.001	0.018	0.031	0.018
Red	0.918	0.004	0.015	0.027	−0.003	0.930	<0.001	0.020	0.032	0.015
NIR	0.961	0.016	0.020	0.036	−0.006	0.856	<0.001	0.023	0.034	0.014
SWIR1	0.932	0.007	0.018	0.031	−0.007	0.893	<0.001	0.021	0.033	0.013
SWIR2	0.946	0.010	0.020	0.032	−0.005	0.909	<0.001	0.021	0.033	0.012
**Date**	**13 September 2011**					**22 October 2013**				
**Parameters**	**R**	**Var**	**MAD**	**RMSE**	**Bias**	**R**	**Var**	**MAD**	**RMSE**	**Bias**
Blue	0.735	0.002	0.012	0.041	0.004	0.980	<0.001	0.006	0.010	0.001
Green	0.902	<0.001	0.015	0.026	0.005	0.985	<0.001	0.007	0.014	0.008
Red	0.904	<0.001	0.017	0.030	0.004	0.986	<0.001	0.008	0.017	0.011
NIR	0.839	0.003	0.034	0.051	0.001	0.968	<0.001	0.008	0.017	0.009
SWIR1	0.868	<0.001	0.022	0.034	0.004	0.979	<0.001	0.009	0.018	0.010
SWIR2	0.903	<0.001	0.023	0.037	0.010	0.986	<0.001	0.009	0.019	0.011

## 4. Discussion

### 4.1. Comparison to STDFA

**Figure 5:**
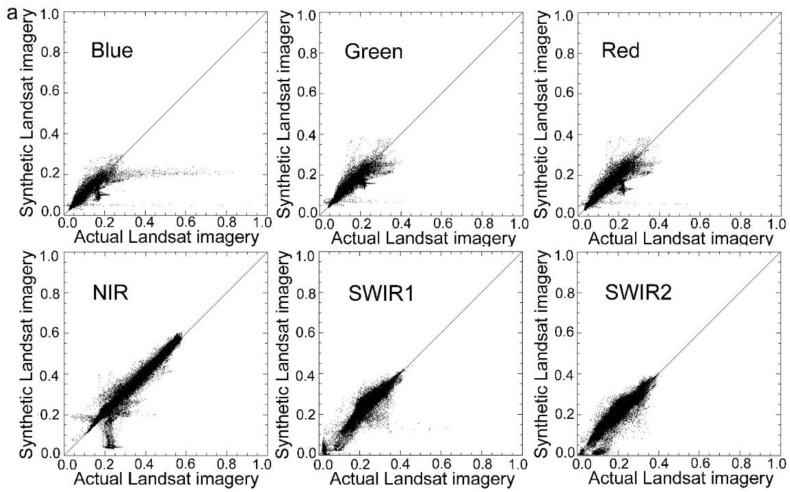

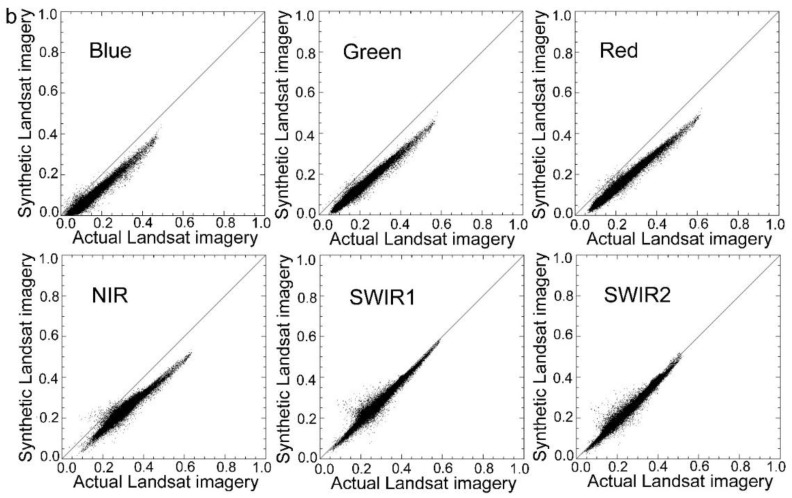
Scatter plots between actual and synthetic images of Landsat in (**a**) Bole and (**b**) Luntai by using the spatial and temporal data fusion approach (STDFA).

Table 6 and Figure 5 show the results of the conditional STDFA. It is evident that in the Luntai area, MSTDFA had better accuracy than the conditional STDFA in nearly all parameters. In the Bole area, MSTDFA had much better accuracy than the conditional STDFA in SWIR1 and SWIR2 bands. However, the precision of MSTDFA was slightly lower in the blue, green, and red bands. A comparison of Figure 4 and Figure 5 revealed that in the Luntai area, the scatter diagram of MSTDFA was closer to the 1:1 line than that of STDFA. Two factors can explain these results. Firstly, MSTDFA has two important improvements over STDFA. In particular, the differences in sensor systems are considered in MSTDFA. In Figure 5, a high correlation is shown between the synthetic and actual Landsat images. However, a certain deviation appeared between the regression line and the 1:1 line that was caused mainly by differences in the sensor systems. Therefore, these deviations were eliminated in the scatter plots of MSTDFA. Secondly, many plots indicate that land cover type changed in the Bole area from 11 to 27 July 2011. For example, the northwest corner of the study area is Aibi Lake which is a huge shallow lake. In addition, Boertala River flows east from the west of the study area. As the water level changed, a lot of bare land changed into water. These changes in land cover type will reduce the precision of the model, and they had a more severe influence for MSTDFA. In STDFA, two days of Landsat images were used to detect the land cover change areas. The land cover change areas were classified as other class. However, only one Landsat image was used in MSTDFA and the change area cannot be detected. So, the land cover type change leads to more reflectance changes of blue, green, and red bands. Thus, MSTDFA performed worse than STDFA in these bands. Details of the influence of land cover change are described in Section 4.5.

**Table 6:** Results of correlation analysis between synthetic and actual Landsat imageries by using the spatial and temporal data fusion approach (STDFA).

Study Area	Bole					Luntai				
Date	27 July 2011					4 September 2013				
Parameters	R	Var	MAD	RMSE	Bias	R	Var	MAD	RMSE	Bias
Blue	0.654	0.002	0.010	0.043	−0.001	0.912	<0.001	0.018	0.035	0.025
Green	0.919	<0.001	0.011	0.021	−0.002	0.922	<0.001	0.018	0.030	0.016
Red	0.930	0.001	0.013	0.024	−0.002	0.930	<0.001	0.020	0.031	0.014
NIR	0.961	0.001	0.020	0.035	−0.006	0.864	<0.001	0.022	0.035	0.016
SWIR1	0.894	0.001	0.019	0.037	−0.003	0.872	<0.001	0.022	0.038	0.020
SWIR2	0.889	0.002	0.024	0.047	0.001	0.892	<0.001	0.024	0.038	0.018
**Date**	**27 July 2011**					**22 October 2013**				
**Parameters**	**R**	**Var**	**MAD**	**RMSE**	**Bias**	**R**	**Var**	**MAD**	**RMSE**	**Bias**
Blue	0.735	0.002	0.013	0.041	0.002	0.968	<0.001	0.010	0.060	0.058
Green	0.907	<0.001	0.016	0.026	0.004	0.974	<0.001	0.011	0.070	0.068
Red	0.899	<0.001	0.019	0.030	0.001	0.977	<0.001	0.012	0.071	0.069
NIR	0.769	0.004	0.041	0.062	0.002	0.956	<0.001	0.011	0.069	0.066
SWIR1	0.863	0.001	0.023	0.035	−0.001	0.978	<0.001	0.010	0.020	0.010
SWIR2	0.894	0.001	0.025	0.038	0.002	0.956	<0.001	0.007	0.026	0.025

### 4.2. Improvement

Compared with traditional STDFA, MSTDFA has two improvements.

Firstly, in the traditional STDFA, the best window size for the solution of Equation (1) was set to a fixed value of 40 coarse pixels. However, owing to the different spatial distribution of each land cover class, the best window size for each land cover class may be different. Thus, a fixed window size may not be appropriate for all classes. In MSTDFA, an adaptive window size and moving step length selection method was used to avoid this problem. This method tested every window size and moved the step length from minimum to maximum by using the exhaustive method, and the correlation coefficient between the reference Landsat mean reflectance and the MODIS disaggregated mean coarse reflectance of every test was calculated. The best window size and moving step length were designed to be those which can lead to the maximum correlation coefficient between the fine and coarse mean reflectance.

Secondly, the differences in sensor systems are not considered in traditional STDFA. In MSTDFA, the sensor differences were removed by using linear models between the Landsat mean reflectance and the disaggregated mean reflectance.

To show the improvements by the above steps, we tested those methods step by step in Luntai at NIR band. Firstly, the basic STDFA model with a fixed window size of 40 MODIS pixels and without sensor difference adjustment was used to generate a synthetic NIR image. Then the adaptive window size selection method and sensor difference adjustment were added step by step to generate a synthetic NIR image. Finally, the similarity between those synthetic NIR imageries and the actual Landsat NIR image were evaluated by correlation analysis. Table 7 shows the results of correlation analysis. From Table 7, we can see improvements of correlation coefficient *R* and declines of *Variance*, *RMSE*, *MAD*, and *bias* in each step. So, the traditional STDFA methods were enhanced by those improvements.

**Table 7:** Accuracy improvements of every step in MSTDFA method of NIR band in Luntai.

Parameters	STDFA	Adaptive Window Size Selection	Sensor Adjustment	Total
R	0.9557	+0.0116	+0.0003	0.9676
Variance	0.0003	−0.0001	0.0000	0.0002
MAD	0.0113	−0.0027	−0.0003	0.0083
RMSE	0.0688	−0.0502	−0.0013	0.0173
Bias	0.0664	−0.0548	−0.0021	0.0095

### 4.3. Landsat and MODIS Fusion Using FROM–GLC Data

**Figure 6:**
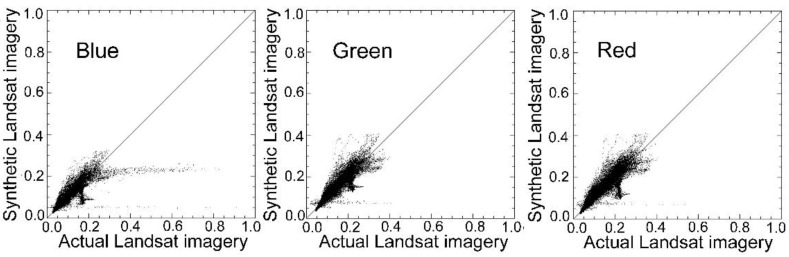

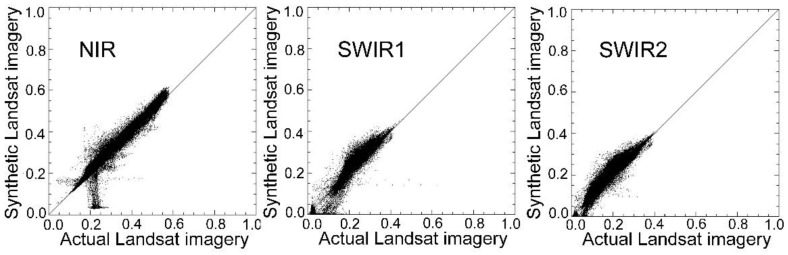
Scatter plots between the actual and synthetic imageries of Landsat using modified spatial and temporal data fusion approach (MSTDFA) with input of Finer Resolution Observation and Monitoring of Global Land Cover (FROM–GLC).

To determine the applicability of the FROM–GLC data in MSTDFA, the FROM–GLC data in Bole mapped by using Landsat-5 TM data acquired on 21 July 2009 was used in MSTDFA. By inputting FROM–GLC data rather than classification data mapped using the maximum likelihood classification method, six synthetic Landsat images ware generated. Table 8 shows the results of accuracy assessment of these six synthetic Landsat images. As indicated in Table 8, most synthetic Landsat images had a high correlation with the actual Landsat imageries with coefficient *R* higher than 0.90. Figure 6 shows the scatter plots between the actual and synthetic Landsat images, which were close to the 1:1 line. These results indicate a high similarity between the actual and synthetic Landsat data and that MSTDFA has high accuracy in generating synthetic Landsat images. Therefore, the FROM–GLC data can be used in MSTDFA.

**Table 8:** Results of correlation analysis between synthetic and actual Landsat imageries using the modified spatial and temporal data fusion approach (MSTDFA) with input of Finer Resolution Observation and Monitoring of Global Land Cover (FROM–GLC) data.

Parameters	R	Variance	MAD	RMSE	Bias
Blue	0.661	0.002	0.011	0.043	−0.003
Green	0.917	0.000	0.012	0.022	−0.003
Red	0.924	0.001	0.014	0.026	−0.004
NIR	0.961	0.001	0.021	0.036	−0.007
SWIR1	0.936	0.001	0.017	0.030	−0.010
SWIR2	0.956	0.001	0.019	0.029	−0.008

### 4.4. Influence of the Image Extents

**Figure 7:**
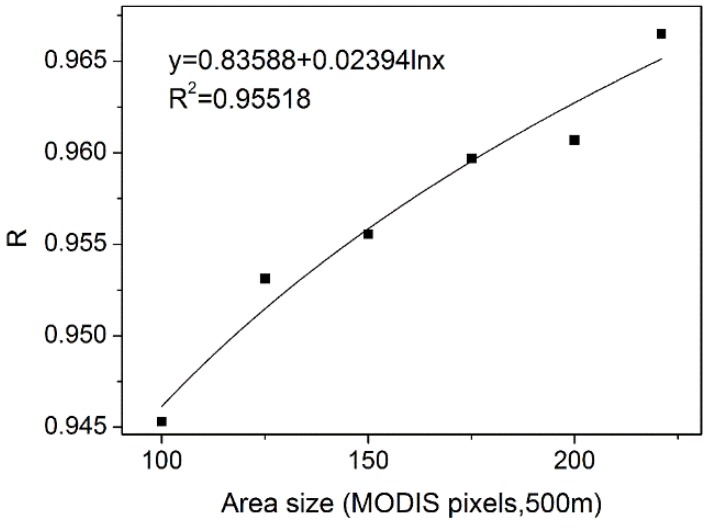
Relationship between correlation coefficient *R* and study area size.

The Ordinary Least Squares technique was used to disaggregate the time series mean surface reflectance value ${\bar{r}}_{d}\left(c,t_{i}\right)$ for each class. However, the solutions will affect the number of pixels an image contains from two aspects. It is easier to generate outliers in the solution of Ordinary Least Squares in a small area. In addition, the images with different extents will produce different disaggregated mean reflectance. These effects were reduced by three steps in this method. Firstly, abnormal disaggregated mean surface reflectance value was not used. Then, all the normal disaggregated mean reflectance for every target MODIS pixels was used to adjust the sensor difference. Finally, the sensor adjusted time series mean fine reflectance ${\bar{r}}_{f}(c,t_{i},m_{k})$ of target MODIS pixels *m_k_* was only used for the fusion of fine pixels belonging to target MODIS pixels *m_k_*. To evaluate the influence of the image extents, we applied MSTDFA in different image extents in NIR band acquired on 22 October 2013 in Luntai. Figure 7 shows that the correlation coefficient *R* has a logarithmic relationship with the size of applied area. The bigger of the study area, the higher the correlation coefficient *R*. So, we recommend using the MSTARFM model in a large area and using the MSTDFA model in areas greater than 200 × 200 MODIS pixels.

### 4.5. Comparison of Actual NDVI and NDVI Calculated Using Synthetic Data

This method was applied to MODIS and Landsat NDVI data in Bole to test its applicability with different data. The MODIS NDVI data included 250 m 16-day MOD13Q1 products acquired on 12 July 2011 and 28 July 2011. The Landsat NDVI data was calculated by using the red and NIR bands of Landsat data acquired on 11 July 2011. By inputting these NDVI data and the land cover data mapped by using the maximum likelihood classification method, a synthetic Landsat NDVI image was generated by using MSTDFA and conditional STDFA. Table 9 shows the accuracy assessment results of the two synthetic Landsat NDVI images. Both STDFA and MSTDFA can be used to fuse NDVI data, and the results of the latter were better than those of the former. Another method to generate synthetic NDVI data is to calculate NDVI using the synthetic red and NIR data generated by STDFA and MSTDFA. Wu *et al.* [41] compared the two methods in the generation of synthetic NDVI and leaf area index.

**Table 9:** Accuracy assessment result comparison of Landsat Normalized Different Vegetation Index (NDVI) fusion application.

	NDVI Generated by MSTDFA	NDVI Generated by STDFA
R	0.970	0.949
Variance	0.006	0.010
MAD	0.043	0.074
RMSE	0.086	0.125
Bias	0.037	0.073

### 4.6. Limitations of the Method

Although MSTDFA can generate daily synthetic Landsat images with high similarity to actual Landsat images, this method has weaknesses.

Firstly, all the spatial and temporal data fusion methods are based on the assumption that the land cover classes do not change over time. Weng *et al.* [28] found that the accuracy of those models will be substantially reduced if this assumption is violated. As discussed in Section 4.2, many plots include land cover changes over time in the Bole area. For example, the northwest corner of the study area is Aibi Lake which is a huge shallow lake. In addition, Boertala River flows east from the west of the study area. As the water level changes, a lot of bare land changes into water. To evaluate the influence of land cover change, we extracted the land change plots by using the differences in Landsat images acquired on 11 July 2011 and 27 July 2011. The residual image between the synthetic and actual images was also calculated. Then, we calculated the correlation coefficient between these two data sets to obtain a high correlation coefficient *R* of 0.992. We also found that pixels with large errors were located in the plots in which the land cover types changed. Therefore, land cover class change has a very important influence in MSTDFA. To assess the influence, we masked these plots and calculated the accuracy assessment parameters again, as shown in Table 10. A comparison of Table 5 and Table 10 revealed that the maximum fusion accuracy improvement was 0.32 in correlation coefficient *R* if the plots are not considered.

**Table 10:** Accuracy assessment result with no consideration of land cover change plots.

	R	Variance	MAD	RMSE	Bias
Blue	0.974	<0.001	0.005	0.011	−0.001
Green	0.989	<0.001	0.006	0.010	−0.002
Red	0.988	<0.001	0.008	0.012	−0.001
NIR	0.997	<0.001	0.009	0.014	−0.004
SWIR1	0.990	<0.001	0.010	0.017	−0.005
SWIR2	0.986	<0.001	0.012	0.019	−0.004

Secondly, this method considered only the fusion of multi-sensor optical images. Therefore, it cannot provide effective data under cloudy conditions because all optical satellites are affected by clouds. In this situation, radar satellite must be considered as a solution [43,44,45,46,47,48].

Thirdly, a simple method in which solutions out of the range of 0%–100% were not used was employed to satisfy the constraints of the solutions of the linear mixed model. Optimization algorithms, such as the normalization algorithm which uses all solutions, minimizing the squared errors between the predicted response variable and observed data, can be used to improve this method [49]. Furthermore, nonlinear least squares regressions for spectral quantitative analysis is also an important direction to modify this method for future research [50].

## 5. Conclusions

In this study, an MSTDFA was developed and validated for two study areas located in Xinjiang, China. By inputting MODIS reflectance data, Landsat data, and land cover data, this method is able to generate daily synthetic Landsat images in which the spatial resolution is the same as that of the Landsat data and the temporal resolution is the same as that of the MODIS data. A comparison with the actual Landsat image revealed the following findings:

(1) The adaptive window size and moving step selection method can select the best window size for disaggregation of coarse pixels. The disaggregated mean coarse reflectance had a strong linear relationship with the Landsat mean reflectance.
(2) MSTDFA had higher accuracy than STDFA but was more easily influenced by land cover change. Land cover data such as that of FROM-GLC can be used in MSTDFA. Synthetic Landsat images with high similarity to actual Landsat images with a correlation coefficient *R* of 0.96 can be generated.
(3) Land cover class change had a very important influence in MSTDFA, which can lead to a reduction in the correlation coefficient *R* of 0.32 in the blue band.
(4) MSTDFA can be applied in 250 m 16-day MODIS MOD13Q1 products and Landsat NDVI data. A synthetic NDVI image with very high similarity to the actual NDVI observation with a high correlation coefficient *R* of 0.97 can be generated.

## Abbreviations

MSTDFA	Modified Spatial and Temporal Data Fusion Approach
MODIS	Moderate Resolution Imaging Spectroradiometer
STDFA	Spatial and Temporal Data Fusion Approach
NDVI	Normalized Different Vegetation Index
AVHRR	Advanced Very High Resolution Radiometer
SPOT	Systeme Pour l’Observation de la Terre
VGT	Vegetation
TM	Thematic Mapper
ETM+	Enhanced Thematic Mapper Plus
OLI	Operational Land Imager
STARFM	Spatial and Temporal Adaptive Reflectance Fusion Model
STAARCH	Spatial Temporal Adaptive Algorithm for mapping Reflectance Change
ESTARFM	Enhanced Spatial and Temporal Adaptive Reflectance Fusion Model
FROM-GLC	Finer Resolution Observation and Monitoring of Global Land Cover
HJ	Huanjing
CCD	Charge Coupled Device
GF-1	Gaofen satellite No. 1
WFV	Wide Field of View camera
USGS	United States Geological Survey
FLAASH	Fast Line-of-Sight Atmospheric Analysis of Spectral Hypercubes
GCPs	Ground Control Points
MRT	MODIS Reprojection Tool
NLCD	National Land Cover Database
NIR	Near Infrared Reflection
SWIR	shortwave infrared
MAD	Mean Absolute Difference
RMSE	Root Mean Square Error
